# Covalently Grafted Peptides to Decellularized Pericardium: Modulation of Surface Density

**DOI:** 10.3390/ijms24032932

**Published:** 2023-02-02

**Authors:** Leonardo Cassari, Martina Todesco, Annj Zamuner, Saima Jalil Imran, Martina Casarin, Deborah Sandrin, Joaquin Ródenas-Rochina, José Luis Gomez Ribelles, Filippo Romanato, Andrea Bagno, Gino Gerosa, Monica Dettin

**Affiliations:** 1 Department of Industrial Engineering, University of Padua, 35131 Padua, Italy; 2 LIFELAB Program, Consorzio di Ricercar Sanitaria, CORIS, Veneto Region, 35128 Padua, Italy; 3 Department of Civil, Environmental and Architectural Engineering, University of Padua, 35131 Padua, Italy; 4 Department of Cardiac, Thoracic and Vascular Sciences, University of Padua, 35128 Padua, Italy; 5 Department of Surgery, Oncology and Gastroenterology, Giustiniani 2, 35124 Padua, Italy; 6 Department of Physics and Astronomy “G. Galilei”, University of Padua, 35131 Padua, Italy; 7 Center for Biomaterials and Tissue Engineering, CBIT, Universitat Politecnica de Valencia, 46022 Valencia, Spain; 8 Biomedical Research Networking Centre on Bioengineering, Biomaterials and Nanomedicine (CIBER-BBN), 46022 Valencia, Spain

**Keywords:** decellularized bovine pericardium, REDV, peptide grafting, peptide surface density, endothelization, HUVECs

## Abstract

The covalent functionalization of synthetic peptides allows the modification of different biomaterials (metallic, polymeric, and ceramic), which are enriched with biologically active sequences to guide cell behavior. Recently, this strategy has also been applied to decellularized biological matrices. In this study, the covalent anchorage of a synthetic peptide (REDV) to a pericardial matrix decellularized via Schiff base is realized starting from concentrated peptide solutions (10^−4^ M and 10^−3^ M). The use of a labeled peptide demonstrated that as the concentration of the working solution increased, the surface density of the anchored peptide increased as well. These data are essential to pinpointing the concentration window in which the peptide promotes the desired cellular activity. The matrices were extensively characterized by Water Contact Angle (WCA) analysis, Differential Scanning Calorimetry (DSC) analysis, geometric feature evaluation, biomechanical tests, and preliminary in vitro bioassays.

## 1. Introduction

Expanding the horn of plenty of biomaterials for soft tissue and organ repair has become a critical issue for tissue engineering. While biological tissues are the gold standard for implantation, research on biomimetic tissue constructs using peptides as constituents of the materials is opening the way to novel improvements. Among biological tissues of xenogeneic origin, decellularized bovine pericardium is used extensively in applications including bioprosthetic heart valves, vascular patches, staple line reinforcement, hernia repair, pelvic floor reconstruction, and bone and dental membranes. Generally, medical devices such as vascular grafts and prosthetic heart valves are scarcely endothelialized, and more often than not, the process results in a partial and fragile cell lining, which may produce thrombotic formations and prosthetic failure. In general, strategies such as in vitro cell seeding produced unsatisfactory results in overcoming the pericardium’s poor adhesion proneness [1,2,3,4,5].

In a previous article, we demonstrated that the tetrapeptide arginine-glutamic acid-aspartic acid-valine (REDV) selective grafting to Decellularized Bovine Pericardium, DBP, allows the in vitro promotion of earlier endothelial cell adhesion and proliferation [6]. The covalent bonding of the REDV sequence to the pericardium was realized through an imine bond between an aldehyde group, appositely inserted after a spacer at the N-terminus of the peptide, and the amines of the pericardium collagen. The imine bond was successively reduced to avoid the reverse reaction. This strategy was previously applied with success to the chemoselective ligation of bioactive peptides and proteins [7,8] and the covalent conjugation of BMP-2 peptides to biomaterials [9,10]. This chemistry, when applied to the pericardium, assured minimal tissue manipulation, as confirmed by the preservation of the structure of natural matrices and of their biomechanical properties, and improved the scaffold’s capacity to promote human umbilical vein endothelial cells’ (HUVECs’) adhesion. In the study of Dal Sasso et al., the REDV concentrations in the anchoring reaction were 10^−6^ M and 10^−5^ M, and the effective surface peptide density, or, in other words, the quantity of anchored REDV peptide, ranged from 10^−15^ to 10^−13^ mol/cm^2^.

These encouraging results prompted us to explore other surface densities of the REDV peptide. It is well known that cells are able to respond to concentrations of adhesive peptides in the order of pmoles/cm^2^ or fmols/cm^2^ [11,12] and that the concentration capable of maximizing cell adhesion may be different from that of the peptide itself when considering the promotion of cellular proliferation [13]. Nevertheless, it is important to experimentally establish the anchored peptide concentration window capable of maximizing the desired biological function (i.e., cell adhesion, proliferation, or differentiation). The methods that allow the covalent anchoring of the peptides to the biomaterial do not always permit a modulation or an easy change of the surface peptide densities; for example, the scarcity of the functional groups on the surface of the material can cause the achievement of very low surface peptide densities. In any case, in the paper of Dal Sasso et al., the highest working concentration of the REDV peptide (10^−5^ M) gave a bound peptide concentration value of 1.17 ± 0.37 10^−13^ moles/cm^2^ for the serosa side and 1.01 ± 0.50 10^−13^ moles/cm^2^ for the fibrosa side of bovine pericardium. Both surface concentrations are below the pmoles/cm^2^ threshold. For this reason, this study aims at evaluating whether: (i) the proposed method can provide functionalized pericardium with higher peptide concentrations; (ii) higher surface concentrations are still biologically active.

The REDV tetrapeptide is present in the sequence of the type III connecting segment of human plasma fibronectin [14] and has been demonstrated to be one of the main sites for the recognition of α4β1 subunits of integrin [15]. The binding between integrins and REDV leads to the selective adhesion of endothelial cells to fibronectin [16,17]. REDV selectively attracts endothelial cells and induces them to proliferate rather than smooth muscle cells (SMCs), fibroblasts, and platelets [16]. Considering its bioactivity and specificity, REDV has gained much attention in the functionalization of biomaterials [16,17,18,19,20,21]. 

The challenge of this work is to demonstrate that the surface concentration can be modified by changing the reaction conditions. The extensive surface characterization was carried out to exclude significant alteration of the geometrical, biomechanical, and chemical-physical features of decellularized pericardial tissue induced by the chemical treatment. A preliminary bioassay was carried out in order to verify the possible cytotoxic effect of functionalized tissues; no adverse effect was shown after 14 days. 

## 2. Results and Discussion

### 2.1. Determination of Surface REDV Density

**Figure 1:**
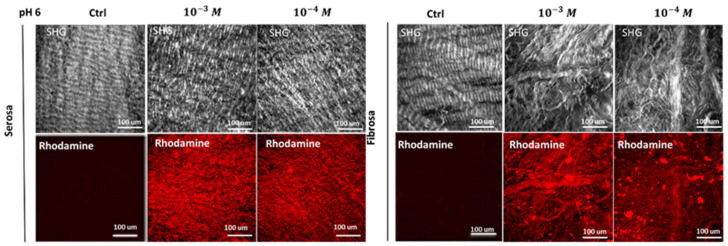
Determination of surface peptide density. The concentrations of REDV (10^−4^ M or 10^−3^ M) solution used to functionalize DBP scaffolds have produced different final quantities of peptides bound to the tissues.

The determination of grafted peptides on the differently treated patches of pericardium, was reached through the Two-Photon Microscope (TPM) measurement of the fluorescence of the Rhodamine probe grafted to REDV sequence. The increase in red intensity is detectable, going from 10^−4^ M to 10^−3^ M in the REDV working solution (Figure 1). The intensity of fluorescence was converted to a number of probes using the regression curve reported in Supplementary Materials (Figure S1). 

Table 1 depicts the values of peptide surface density values for all samples including those obtained in our previous paper ([6]; 10^−5^ M or 10^−6^ M REDV working solutions) for an easier comparison. There is an increase from 8.14 ± 1.77 × 10^−16^ moles/cm^2^ (treatment with 10^−6^ M REDV working solution) to 46.71 ± 8.07 × 10^−12^ moles/cm^2^ (treatment with 10^−3^ M REDV working solution) in the REDV surface density for the serosa side, and an increase from 1.00 ± 0.62 × 10^−15^ moles/cm^2^ (treatment with 10^−6^ M working solution) to 34.22 ± 15.43 × 10^−12^ moles/cm^2^ (considering the fibrosa side) in the REDV surface density for the fibrosa side. The improvement in peptide surface density (moles/cm^2^) is more than times for the serosa side and about times for the fibrosa side due to an increment of 1000 in the working peptide concentration. The difference in the amounts of the increments reflects the different composition and roughness (effective exposed area) of the two sides of the pericardium.

The proposed anchoring method has several advantages, including: (i) no organic solvent or condensing agent is used: for example, the conjugation of bioactive peptides to biological matrices through 1-Ethyl-3-(3-Dimethylaminopropyl)Carbodiimide (EDC) carboxylic acid pre-activation could entail EDC-mediated cross-linking of collagen [22]; (ii) specific and selective bond formation between the bioactive sequence and the matrix: the addition of the aldehyde moiety to the peptide sequence assures the selectivity and specificity of the bond and avoids the involvement of peptide side-chain into the anchorage; the side chain groups are available for interaction with cell receptors to mediate cell adhesion; (iii) the change of peptide solution concentration determines the grafted peptide concentration on the scaffold: consequently, it is likely to identify the grafted peptide concentration window able to promote endothelial cell adhesion.

**Figure 2:**
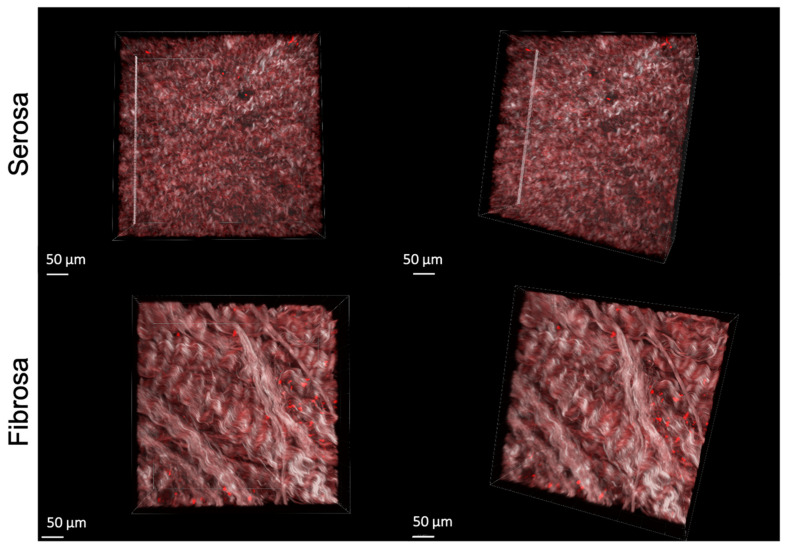
3D representation of RhodREDV functionalized pericardium. The images were obtained by overlapping the Second Harmonic Generation (SHG) signal relative to collagen and the RhodREDV signal at 800 nm.

Figure 2 illustrates the 3D reconstruction of the serosa and fibrosa layers of DBP functionalized with 10^−4^ M of REDV peptide. 

### 2.2. WCA and DSC Measurements

**Figure 3:**
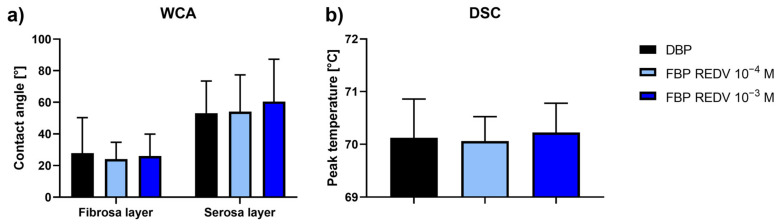
WCA and DSC analyses. (**a**) Water contact angle measurements on the functionalized serosa and fibrosa sides of DBP (functionalized bovine pericardium, FBP); (**b**) differential scanning calorimetry (DSC) peak temperature of functionalized DBPs in the hydrated state recorded at 20 °C/min between 25 °C and 90 °C.

The results of WCA measurements on both the serosa and fibrosa sides of the samples show no significant difference between functionalized samples (working solutions: 10^−4^ M and 10^−3^ M) versus untreated pericardium (Figure 3a).

The DSC determination carried out for functionalized DBP samples (Figure 3b) gave values comparable with those of not functionalized samples (the control).

These results confirm the preservation of both surface hydrophilicity and collagen structure within pericardial patches.

### 2.3. Biomechanical Characterization

**Figure 4:**
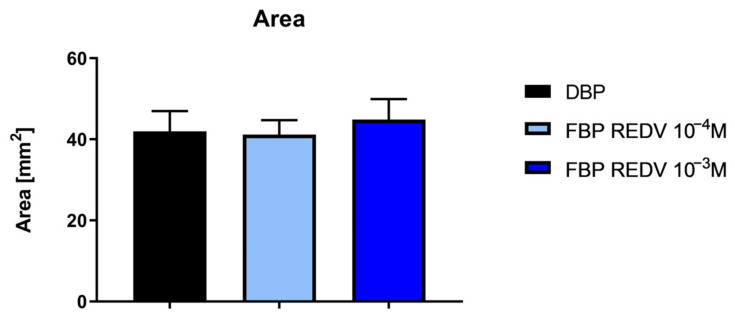
Measurement of the area of functionalized and not functionalized samples.

An evaluation of the sample area is reported in Figure 4. The functionalization treatment does not induce any significant modification of the sample area.

**Figure 5:**
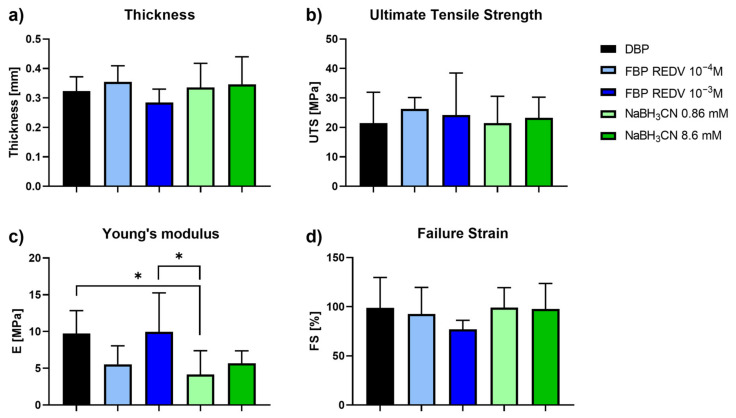
Mechanical tests performed on functionalized and not functionalized samples. (**a**) Evaluation of DBP samples’ thickness; (**b**) Ultimate Tensile Strength; (**c**) Young’s modulus; and (**d**) Failure Strain values obtained on DBP and functionalized DBP samples. * *p*-value < 0.05.

Figure 5 reports the results of mechanical tests performed on functionalized and non-functionalized samples. Two other conditions were considered: the pericardium samples were treated with two different concentrations of the reduction agent (NaBH_3_CN). These are controls for the functionalized samples at 10^−4^ M and 10^−3^ M concentrations of the peptide REDV (working concentrations), respectively.

Interestingly, the thickness, the failure strain, the ultimate tensile strength, and Young’s modulus (Figure 5c) did not show any significant variation between DBP and functionalized bovine pericardium (FBP REDV 10^−4^ M and FBP REDV 10^−3^ M). Indeed, with regard to the Young’s modulus, a significant difference appeared between DBP and FBP REDV 10^−3^ M if compared with NaBH_3_CN 0.86 mM, *p* = 0.0154 and *p* = 0.011, respectively. 

In our opinion, the significant difference between DBP and NaBH_3_CN 0.86 mM DBP is attributed to the reduction of naturally occurring crosslinks in collagen involving ε-NH_2_ of Lys and Lys aldehyde. The reduction agent affects 10^−4^ M REDV-treated samples, 0.86 M NaBH_3_CN samples, and 8.6 mM samples with respect to the untreated samples (DBP), and produces matrices less stiff than the control. One exception is the DBP treated with a 10^−3^ M REDV solution; we are speculating that a high REDV concentration could have a protective action toward the reduction of interchain Schiff bases.

### 2.4. Immunofluorescence Staining

**Figure 6:**
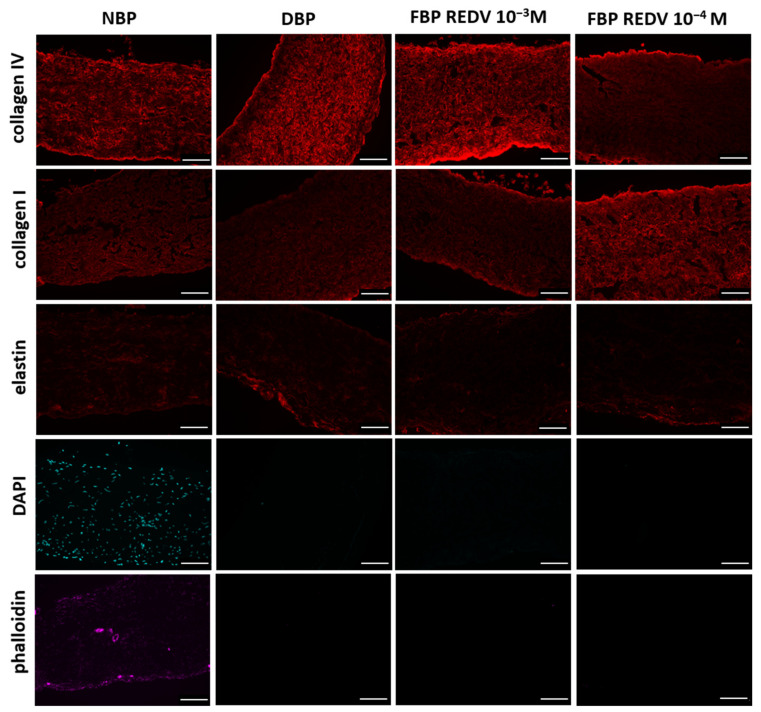
Immunofluorescence staining rows report the specific staining, while columns show the specific tissue analyzed (scale bar = 100 μm).

Figure 6 shows the microscopic structure of NBP and DBP in comparison with two different concentrations of functionalized, decellularized bovine pericardium, FBP REDV 10^−3^ M and FBP REDV 10^−4^ M. 

When compared to the native tissue, immunostaining shows that the functionalization process had no effect on ECM architectural proteins such as collagen I, collagen IV, and elastin in DBP and functionalized tissue at both concentrations. As demonstrated in Figure 6, the pericardium is mainly composed of collagen, which maintains the typical wavy pattern even after decellularization and functionalization processes. Both procedures did not damage fibers. Elastin, whose pericardium is extremely poor, did not show a further loss after decellularization and functionalization. 

The absence of nuclei and actin, counterstained with DAPI and phalloidin, respectively, confirms the efficacy of the decellularization method applied in the present study.

Direct contact seeding of HUVECs was performed according to ISO 10993 part 5 [23] to assess the cytocompatibility of the functionalized tissue. A live and dead assay was realized by staining live cells in green and dead cells in red to evaluate the cytotoxicity and the morphological organization of seeded cells on tissue.

**Figure 7:**
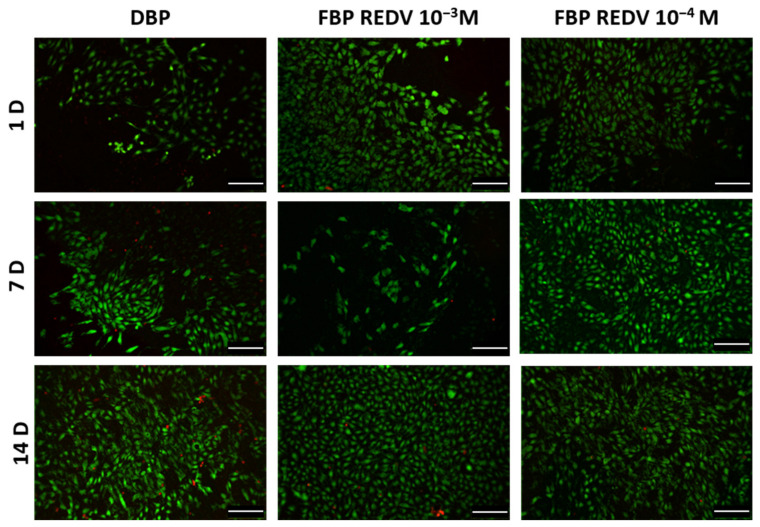
Live/dead staining on seeded samples of DBP and functionalized tissues (columns) analyzed at days 1, 7, and 14 (rows). Scale bar = 100 μm.

As shown in Figure 7, cell viability is higher in both functionalized samples, FBP REDV 10^−4^ M and FBP REDV 10^−3^ M, as demonstrated by the lower number of dead cells with respect to the DBP control at all time points. Moreover, the live cell number increased after 7 and 14 days in DBP and FBP REDV 10^−4^ M, while in the case of FBP REDV 10^−3^ M, after a decrease at day 7, significant cell growth was detected. In all the experimental groups, HUVECs maintained their typical rounded shape.

A quantification of the percentage of viable cells (mean ± standard deviation) for each experimental condition is reported in Table 2.

Regarding DBP, a significant difference was detected in the number of viable cells between 24 h and 14 days, while in the case of FBP REDV 10^−3^ M, a significant difference was found between 24 h and both 7 and 14 days. In the case of FBP REDV 10^−4^ M, however, no significant differences were discovered. 

At the same time point, a significant increase of viable cells was found between DBP and FBP REDV 10^−4^ M at day 7 and between DBP and FBP REDV 10^−3^ M at day 14. 

Looking at Table 2, the FBP REDV 10^−4^ M samples showed higher cell viability both at 7 and 14 days from the seeding, although the live-and-dead assay (Figure 7) showed a lower number of HUVECs adhered to FBP REDV 10^−4^ M than both DBP and FBP REDV 10^−3^ M at 7 days from the seeding.

## 3. Materials and Methods

### 3.1. Materials

All amino acids used for the peptide synthesis were supplied by Novabiochem (Merck KGaA, Darmstadt, Germany). All other materials were supplied by Sigma-Aldrich (Saint Louis, MO, USA), unless otherwise stated.

### 3.2. TergiCol Decellularization and Sterilization

Native (fresh) Bovine Pericardium (NBP) was harvested from a local slaughterhouse and treated within 3 h from healthy animals (Holstein Friesian calves, 7 months old, weighing between 300 and 350 kg) in accordance with European Commission (EC) Regulation 1099/2009 on animal health and protection [24,25,26]. Briefly, the left anterior region of the pericardium was isolated, and the tissues were cleaned by removing the fatty lining. Decellularization was performed according to the TergiCol decellularization procedure: NBP samples were treated with protease inhibitors, hypo- or hyperthermic solutions, and detergents, first Tergitol 0.1–1% *v*/*v*, and then 10 mM sodium cholate. The tissues were finally treated with 10% *v*/*v* isopropanol and washed with saline solution (0.9% *w*/*v*). The tissues were then treated with a nonspecific endonuclease (Benzonase™) to degrade double- and single-stranded nucleic acids [27]. 

Decellularized porcine pericardium (DBP) samples were sterilized with a cocktail of antibiotics, antimycotics, and peracetic acid as described by Fidalgo et al. [28]. 

### 3.3. Evaluation of Decellularized Scaffold Histoarchitecture

Direct and indirect immunofluorescence staining were performed in NBPs, DBPs, and functionalized tissues (FBP REDV 10^−3^ M and FBP REDV 10^−4^ M) in order to assess the effect of decellularization and peptide functionalization on the extracellular matrix (ECM) in the microstructure.

Samples were embedded in optimum cutting temperature (OCT) compound (Tissue-Tek, Alphen Aan den Rijn, The Netherlands) and stored at −80 °C until cryostat cutting. Tissue cryosections (6 μm) were fixed in 4% *w*/*v* ParaFormAldehyde (PFA) provided by Bioptica, and after 2 washes with PBS, the following primary antibodies were used: collagen I (1:100, C2456; Sigma), elastin (1:50, ab21610, Abcam), and collagen IV (1:200, ab6586, Abcam). The controls were incubated with 1% (*w*/*v*) bovine serum albumin instead of primary antibodies. 

The following secondary antibodies were subsequently used: goat anti-mouse Alexa Fluor 555 (1:300, A21422; Invitrogen) and goat anti-rabbit Alexa Fluor 555 (1:300, A27039; Invitrogen). 

Nuclei were directly stained with 4′,6-diamidino-2-phenylIndole (DAPI, Invitrogen, Thermo Fisher Scientific, Waltham, MA, USA), following the producer’s instructions, while filamentous actin was fluorescently labelled with phalloidin–Atto 647N (1:200, 65906, Sigma-Aldrich, St. Louis, MO, USA) [24].

Images were acquired with an epifluorescence microscope, the Leica AF6000, connected to a Leica DC300 digital camera and equipped with LAS AF software (Leica Micro-System, Wetzlar, Germany). Image processing was performed using the open-source Fiji software (NIH, Bethesda, MD, USA). 

### 3.4. Peptide Synthesis

The tetrapeptide arginine-glutamic acid-aspartic acid-valine (REDV) was synthesized in the laboratory using a solid-phase method and an automatic synthesizer (Syro I, MultiSynTech GmbH, Witten, Germany) and fluorenylmethyloxycarbonyl (Fmoc) chemistry [29]. Two spacers (7-aminoeptanoic acid, Ahp) were added to the N-terminus of the REDV sequence before the last insertion of a Ser residue (complete sequence: H-Ser-Ahp-Ahp-Arg-Glu-Asp-Val-NH_2_). The resin used was Rink Amide 4-MethylBenzHydrylAmine (MBHA, 0.52 mmoles/g, Novabiochem, Merck). Fmoc-amino acids were protected at their side chains as follows: pentamethyl-2,3-dihydrobenzofuran-5-sulfonyl (Pbf) for Arg, tert-butyl (OtBu) for Asp and Glu, and t-butyl ether (tBu) for Ser were used. At the end of the synthesis, after Fmoc deprotection, side chain protecting groups were deblocked, and the peptide was cleaved from the resin through a treatment with a solution of H_2_O, triethyl silane, and trifluoroacetic acid (2.5:2.5:95) for 1.5 h at room temperature. The conversion of Ser into ketoaldehyde was carried out by NaIO_4_ treatment for 4 min at room temperature (30 mg of peptide and 8.4 mg NaIO_4_ in 15 mL of water) under stirring. The crude peptide (HCO-CO-NH-Ahp-Ahp-Arg-Glu-Asp-Val-NH_2_) was purified in semipreparative reversed-phase high-performance liquid chromatography (RP-HPLC) as follows: column, Zorbax 300SB C_18_ (5 µm, 300Å, 9.4 × 250 mm); flow rate, 4 mL/min; eluent A, 0.05% trifluoroacetic acid (TFA)/H_2_O; eluent B, 0.05% TFA/CH_3_CN; gradient, 10–30%B over 40 min, detection at 214 nm. The best fractions were collected and lyophilized. The homogeneity of the purified peptide (retention time = 13.874 min) was 99.7%; the datum resulted from the integration of the analytical chromatogram (conditions: column, Vydac 218TP C_18_ (5 µm, 300Å, 9.4 × 250 mm); flow rate, 1 mL/min; eluent A, 0.05% TFA/H_2_O; eluent B, 0.05% TFA/CH_3_CN; gradient, 10–30%B over 20 min, detection at 214 nm. The identity of the peptide was assessed by matrix-assisted laser desorption/ionization (MALDI) mass spectrometry (experimental mass: 827.16 Da; theoretical mass: 826.99 Da, 4800; MALDI-*Time Of Flight* (TOF)/TOF TM analyzer provided with 4000 Series Explorer TM software (Applied Biosystems/MDS Sciex, Foster City, CA, USA)).

The REDV analog is labeled with rhodamine fluorophore and named RhodREDV; its sequence is: H-Ser-Ahp-Ahp-Arg-Glu-Asp-Val-Lys-(TAMRA)-Ahp-NH_2_ (TAMRA = 5(6)-carboxytetramethylrhodamine) was synthesized by the solid-phase method via Fmoc chemistry. The loading of Rink Amide resin (0.52 mmoles/g) was carried out with Fmoc-Ahp-OH (double coupling). The subsequent insertion was performed with Fmoc-N-ε-1- 4,4-Dimethyl-2,6-Dioxocyclohex-1-ylidene)Ethyl-Lys-OH (Fmoc-Lys(Dde)-OH) for introducing a new grade of orthogonality. The introduction in the synthesis of the Ahp spacer (loading) before Lys (Dde) was a cautious move to avoid problems of steric hindrance in the following functionalization of the Lys side chain with TAMRA. The Dde protecting group was removed by treating the peptide on resin with 1.8 mmoles of NH_2_OH•HCl and 1.35 mmoles of imidazole in *N*-*Methyl*-2-pyrrolidone (NMP): CH_2_Cl_2_ (5:1) for 3 h [30]. The resin was washed with N,N-dimethylformamide (DMF) and dichloromethane (DCM) and dried under vacuum for 1 h. The condensation between TAMRA and εNH_2_ of Lys was carried out by adding 8 eq. of TAMRA, 16 eq. of N,N-diisopropylethylamine (DIPEA), and 8 eq. of N,N,N’,N’-tetramethyl-O-(1H-benzotriazol-1-yl)uronium hexafluorophosphate (HBTU)/Ethyl cyano(hydroxyimino)acetate OximaPure (2 h) in DMF. The resin was filtered and washed with DMF and DCM and then dried 1 h under vacuum. The cleavage from the resin and the oxidation of the N-terminal Ser were performed as reported for an unlabeled peptide. The homogeneity of the purified peptide (retention time = 17 and 20 min for the two conjugation products due to the two isomers of rhodamine used) was about 98%: the datum resulted from the integration of analytical chromatogram (Conditions: column, Vydac 218TP C_18_ (5 μm, 300Å, 4.6 × 250 mm); flow rate, 1 mL/min; Eluent A, 0.05% TFA/H_2_O; Eluent B, 0.05% TFA/CH_3_CN; Gradient, 20–35%B over 30 min, detection at 214 nm. The identity of the peptide was checked by MALDI mass spectrometry (experimental mass: 1494.13 Da; theoretical mass: 1494.85 Da).

### 3.5. Functionalization of the Biological Scaffolds

Purified lyophilized peptides were dissolved in PBS at pH 6, at a starting concentration of 10^−3^ M. A reducing agent, i.e., sodium cyanoborohydride (Merck Millipore), was added (2.19 mg of NaBH_3_CN for 1 mg of peptide). The final solution was also diluted to produce the lower concentrations of 10^−4^ M, both in the case of RhodREDV and REDV. Both 10^−4^ M and 10^−3^ M peptide solutions were sterilized with a syringe filter of 0.22 µm porosity. 

DBPs samples were cut into 2 × 2 cm^2^ squared specimens and fixed in custom-made sterile inserts with a culture area of 0.5 cm^2^ under aseptic conditions. Sterilization was done as described above. Each sample was placed into a 24-well plate (Sarstedt, Nümbrecht, Germany) and functionalized with 100 µL of peptide solution for 24 h at room temperature. Samples were then washed 3 times with PBS under gentle agitation. 

### 3.6. Quantification of Functionalization

The yield of functionalization was measured using RhodREDV. Quantification of the RhodREDV peptide covalently linked to functionalized DBPs (n = 3) was performed using a method already reported [31,32] based on TPM-acquired images. The calibration curve was prepared with serial dilutions of free RhodREDV in PBS at pH 6 (Figure S1). The intensity was measured through Fiji [33], considering three regions of interest (ROIs) for each tested sample, respectively. Blank was set with PBS for the calibration curve and with the natural fluorescence of pericardial and aortic ECM proteins for differently treated scaffolds. Effective peptide surface densities were calculated by multiplying the estimated concentrations by the depth of the focal volume (2 µm).

### 3.7. Geometrical Properties of the Tissues

Tissue punches of DBPs were obtained using a biopsy puncher with an 8-mm diameter, and the thicknesses and areas of 10^−4^ M, 10^−3^ M REDV-functionalized, and control scaffolds (n = 9) were measured in order to evaluate whether the functionalization could affect the geometrical properties of the treated tissues. A Mitutoyo digital caliber (model ID-C112XB, Mitutoyo America Co., Aurora, IL, USA) was used to measure samples’ thickness by sandwiching them between two glass slides, whose thickness was then subtracted while surface areas were calculated on sample images by means of the Fiji image processing platform.

### 3.8. Uniaxial Tensile Test

The effect of functionalization treatment on DBP was investigated by means of uniaxial tensile tests. This analysis was performed on DBP and functionalized samples, FBP REDV 10^−4^ M and FBP 10^−3^ M, but also on pericardial samples treated with two different concentrations of the reduction reagent (NaBH_3_CN) as controls for the functionalized samples at 10^−4^ M and 10^−3^ M concentrations of the REDV peptide (working concentrations), respectively. 

After functionalization of rectangular samples of tissues (3 × 3 cm^2^), three dog-bone-shaped specimens were cut for each sample with a custom-made cutter following ASTM D1708-13 [34], and the samples’ thickness was measured as previously described. Uniaxial tensile loading tests were performed at room temperature with a custom-made apparatus (TRAMA, IRS, Padova, Italy) equipped with four linear actuators and four loading cells (50 N). During the test, two opposite actuators were employed, and specimens were preloaded up to 0.1 N and then elongated until rupture with an elongation rate of 0.2 mm/s. 

Collected data on force and elongation were processed using an in-house Matlab^®^ script (Mathworks, Natick, MA, USA) [24,35]. Stress-strain curves were plotted. From these curves, the following parameters were calculated: failure strain (FS) is the maximum elongation achieved by the materials; ultimate tensile strength (UTS) is the maximum strength that the sample can withstand before breaking; and Young’s modulus (E) is the slope of the stress-strain curve in the linear region (1–10%). 

### 3.9. Water Contact Angle (WCA)

The surface wettability was traced by measuring the static WCA. An OCA20 instrument (Dataphysics), equipped with a Charge Coupled Device (CCD) camera for the drop shape analysis, was used at 25 °C and 65% relative humidity. Three μL of ultrapure water were applied to different areas of the samples’ surface. The static contact angles were then measured on both sides of the two-dimensional projection of the droplet by digital image analysis. Data are reported as the average of at least three separate measurements on both the serosa and fibrosa sides of DBPs, with each analysis performed in triplicate (n = 9).

### 3.10. Differential Scanning Calorimetry (DSC)

The phase transition thermograms were recorded with a DSC 8000 differential scanning calorimeter from Perkin Elmer. Temperature and energy scales were calibrated using the manufacturer’s instructions and indium and zinc as standards. Hydrated samples between 5 and 10 mg in weight were sealed in 30 mL aluminum pans. Empty pans were used as references. Thermal analysis was mainly performed to get insight into the denaturation phenomenon of collagen, which is known to occur between 40 and 80 °C in the hydrated state and between 180 and 230 °C in the dehydrated state. For this reason, the investigations were performed between 25 and 90 °C with a 20 °C/min heating rate.

### 3.11. Assessment of REDV-Functionalization Bioactivity and Cytotoxicity

HUVECs’ adhesion was evaluated by means of viability assays. In vitro cytotoxicity tests were carried out according to ISO 10993 Part 5 requirements with a direct contact test assay [23]. Seeded cells’ viability was investigated after 1, 7, and 14 days.

#### 3.11.1. Cell Culture

Human umbilical vein endothelial cells (HUVECs) from a single donor (C-12200, PromoCell GmbH, Heidelberg, Germany) were expanded at 37 °C in a 5% CO_2_ incubator with a humidified atmosphere. Cells were seeded at passage 4 at a density of 30,000 cells/cm^2^ on DBPs and functionalized samples (n = 3) and cultivated in static conditions with endothelial growth medium supplemented with 1% (*v*/*v*) penicillin-streptomycin (PromoCell GmbH, Heidelberg, Germany). Samples were collected on days 1, 7, and 14. Untreated DBPs were considered as the control.

#### 3.11.2. Cell Viability

A qualitative evaluation of cell viability was performed by means of fluorescent Live/Dead staining after 1, 7, and 14 days, according to the manufacturer’s protocol (MP 03224, Thermo Fisher Scientific, Waltham, MA, USA). Briefly, live cells were stained in green by Calcein AM (2 μM), while dead cells were stained in red with by Ethidium homodimer-1 (4 μM). Seeded samples were incubated at 37 °C for 45 min, and at the end of the incubation period, the staining solution was replaced with fresh medium. Epifluorescence images were taken by means of an Olimpus IX71 microscope.

Samples were analyzed by acquiring four ROIs for each sample, which were elaborated with Fiji’s built-in plugins [33]. Live and dead cells were calculated in each ROI and were expressed as a mean ± standard deviation. A statistical ANOVA test was chosen in order to compare the variability over time in each group (24 h as the control group) and, at the specific time point, to compare the different groups (DBP as the control group).

The percentage of viable cells was calculated as follows

(1) $$\%\;\mathrm{viable}\;\mathrm{cells}=\frac{\mathrm{live}\;\mathrm{cells}}{\mathrm{live}\;\mathrm{cells}+\mathrm{dead}\;\mathrm{cells}}\;\cdot \;100$$

#### 3.11.3. Statistical Analysis

Data were processed and analyzed with Prism (GraphPad Software). Results are reported as mean ± standard deviation. The data were statistically compared using variance analysis (ANOVA). A two-way ANOVA with the Bonferroni multiple comparison test was performed to assess the statistical significance of WCA measurements. The significant level was set at 5%.

## 4. Conclusions

Decellularized bovine pericardium is a biological tissue widely used to prepare transcatheter heart valves. The advantage of using xenogenic biological tissues compared to synthetic substitutes lies in their similarity to native tissue in terms of structure, biomechanics, and biological functionality. Furthermore, xenogenic biological tissues can be manipulated and sutured and are easily available. On the other hand, it should always be remembered that a decellularized tissue is also deprived of the endothelium layer, which ensures its antithrombotic properties; in other words, the decellularized tissue is thrombogenic as it is mainly composed of collagen, which, by interacting with the von Willebrand factor and the coagulation protein, promotes platelet adhesion. We have previously demonstrated that covalent functionalization of the bovine pericardium with the peptide REDV promotes faster endothelialization. Recently, the functionalization of arterial and venous grafts with REDV has been successfully tested in a rat model [36], indicating the promising use of decellularized and peptide-functionalized biological materials for in vivo re-endothelialization. In this study, operative concentrations one or two orders of magnitude higher than those previously published were used for the functionalization of the decellularized bovine pericardium. The study demonstrates the possibility of modulating the concentration of anchored REDV from 0.0008 pmoles/cm^2^ up to 47 pmoles/cm^2^ (five orders of magnitude). The composition of the two sides of the biological tissue is quantitatively different, reflecting the different composition and structure. The functionalization treatment is simple and does not induce significant changes in the surface and mechanical properties of the decellularized biological tissue.

Preliminary in vitro bioassays demonstrated the absence of cytotoxicity and that the viability of HUVEC cells is higher in both functionalized samples (10^−4^ and 10^−3^ M REDV) compared to untreated DBP. A quantitative evaluation of HUVEC adhesion and proliferation would be carried out to identify the most performing surface in static and dynamic cell seeding.

## Figures and Tables

**Table 1:** Working REDV concentration and effective REDV surface densities on DBP.

Working Concentration	Surface Density (mol/cm^2^)	
	Serosa	Fibrosa
10^−6^ M REDV	8.14 ± 1.77 × 10^−16^	1.00 ± 0.62 × 10^−15^
10^−5^ M REDV	1.17 ± 0.37 × 10^−13^	1.01 ± 0.05 × 10^−13^
10^−4^ M REDV	14.03 ± 6.14 × 10^−12^	13.60 ± 7.67 × 10^−12^
10^−3^ M REDV	46.71 ± 8.07 × 10^−12^	34.22 ± 15.43 × 10^−12^

**Table 2:** HUVECs viability at 1, 7, and 14 days after seeding on DBP control and REDV-functionalized samples.

Time Point	DBP	10^−4^ M REDV	10^−3^ M REDV
24 h	75.69 ± 24.43%	98.65 ± 1.05%	99.31 ± 0.50%
7 days	83.88 ± 14.67%	98.23 ± 1.63%	97.5 ± 5%
14 days	88.84 ± 4.74%	98.88 ± 0.95%	97.91 ± 1.22%

## Data Availability

Not applicable.

## Supplementary Materials

The following supporting information can be downloaded at: https://www.mdpi.com/article/10.3390/ijms24032932/s1.
