# Supplementary material for: Covalently Grafted Peptides to Decellularized Pericardium: Modulation of Surface Density
Source: Int J Mol Sci. 2023 Feb 2;24(3):2932. doi: 10.3390/ijms24032932 (PMC9917601; doi:10.3390/ijms24032932)
Supplement: Supplementary file 1 [file ijms-24-02932-s001.zip › ijms-2116361-supplementary.pdf]

International Journal of *Molecular Sciences*

MDPI

*Supplementary Materials*

# Covalently Grafted Peptides to Decellularized Pericardium: Modulation of Surface Density

**Leonardo Cassari [1,2#], Martina Todesco [2,3#], Annj Zamuner [2,3], Saima Imran [2,4], Martina Casarin[2,5], Deborah Sandrin [2,6], Joaquin Ródenas-Rochina[7], José Luis Gomez Ribelles [7,8], Filippo Romanato [2,6], Andrea Bagno [1,2], Gino Gerosa [2,4§], Monica Dettin [1,2§*]**

1 Department of Industrial Engineering, University of Padua, Padua, Italy
2 LIFELAB Program, Consorzio di ricercar Sanitaria, CORIS, Veneto Region, Italy
3 Department of Civil, Environmental and Architectural Engineering, University of Padua, Padua, Italy
4 Department of Cardiac, Thoracic and Vascular Sciences, University of Padua, Padua, Italy
5 Department of Surgery, Oncology and Gastroenterology, Giustiniani 2, 35128 Padua, Italy
6 Department of Physics and Astronomy "G. Galilei", University of Padua, Padua, Italy
7 Center for Biomaterials and Tissue Engineering, CBIT, Universitat Politecnica de Valencia, Valencia, Spain
8 Biomedical Research Networking Centre on Bioengineering, Biomaterials and Nanomedicine (CIBER-BBN), Valencia, Spain
# These authors contributed equally to this study
§ These authors contributed equally to this study
* Correspondence: monica.dettin@unipd.it; Tel.: +39 049 8275553

## *1.S Two-Photon Microscope Calibration curve*

A calibration curve was constructed by reading the signals emitted by different Rhod-REDV PBS solutions at pH 6 (10 μL) at concentrations of 100 nM, 1 μM, 10 μM, and 100 μM, respectively and subtracting the background signal of pure PBS (Figure S1).

Three measurements per concentration were performed, considering focal volumes with a 484 μm square base (ROI) and 2 μm height (resolution in z). Eventually, the interpolation (regression) line with its equation has been calculated.

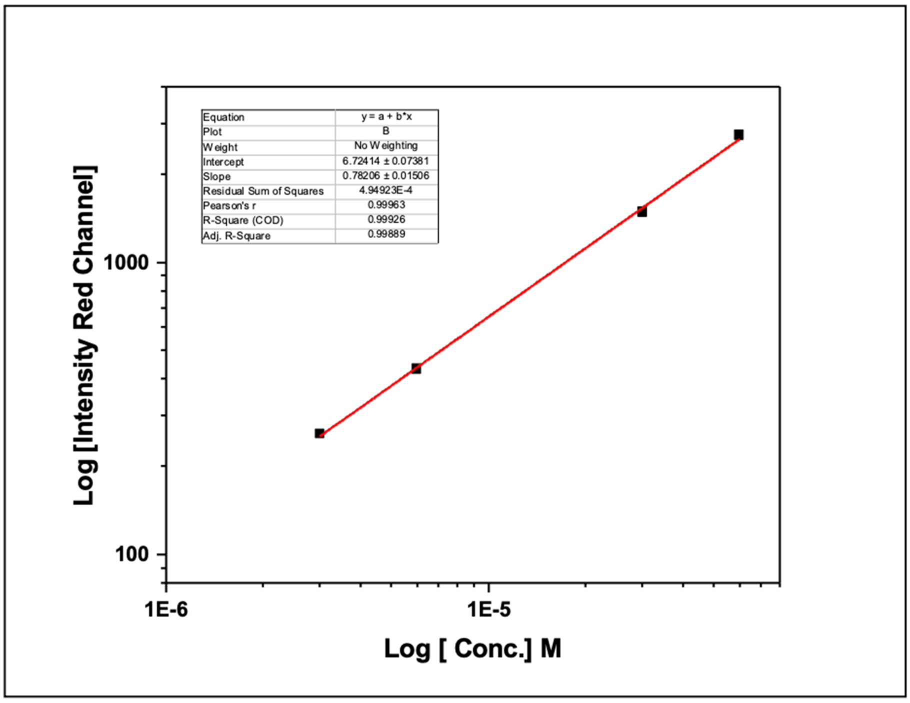


**Figure S1.** TPM calibration curve.
